# When Antiretroviral Therapy Is Not Enough: A Case Series of Pneumocystis Pneumonia With Pulmonary Complications in HIV-Positive Patients

**DOI:** 10.7759/cureus.93015

**Published:** 2025-09-23

**Authors:** Smita V Mohod, Rozina Sheikh, Bhoomika Choudhary, Megha Vijay, Gopal Agrawal

**Affiliations:** 1 Microbiology, Indira Gandhi Government Medical College, Nagpur, Nagpur, IND

**Keywords:** hiv, opportunistic infections, pneumocystis jirovecii, pseudomonas aeruginosa, pulmonary complications

## Abstract

Pulmonary opportunistic infections remain a major cause of morbidity in people living with HIV (PLHIV), especially with poor antiretroviral therapy (ART) adherence or comorbidities. *Pneumocystis jirovecii* pneumonia (PJP) is a frequent and life-threatening infection that may be complicated by bacterial superinfections or post-tubercular sequelae.

We describe two HIV-positive adults with severe pulmonary involvement. The first, a 38-year-old man with interrupted ART, presented with hemoptysis, fever, and oral ulcers. Cluster of differentiation 4 (CD4) count was 80 cells/mm³; sputum showed *P. jirovecii* cysts, and culture grew *Pseudomonas aeruginosa*. High-resolution computed tomography (HRCT) revealed fibrocavitary post-tubercular changes with superinfection. The second, a 55-year-old woman with diabetes and hypertension on ART, developed hypoxemic respiratory failure; sputum confirmed PJP with bilateral infiltrates on imaging. Both improved with high-dose co-trimoxazole, corticosteroids, ART, and supportive care.

These cases highlight the persistent burden of PJP in PLHIV, the risks of poor ART adherence, and the role of timely diagnosis and comprehensive management in improving outcomes.

## Introduction

HIV-associated pulmonary infections remain a significant cause of morbidity and mortality globally, particularly in resource-limited settings [1]. *Pneumocystis jirovecii* pneumonia (PJP) is a common opportunistic infection in people living with HIV (PLHIV), particularly when the cluster of differentiation 4+ (CD4+) T-cell count falls below 200 cells/mm³ [2]. The introduction of antiretroviral therapy (ART) and co-trimoxazole prophylaxis has markedly reduced PJP incidence; however, cases still occur due to late diagnosis, poor adherence, or immune nonresponse to ART.

In tuberculosis (TB)-endemic countries such as India, a substantial proportion of PLHIV have structural lung damage from prior tuberculosis [3]. *Pseudomonas aeruginosa* infection is considered a major cause of morbidity in all patients with bronchiectasis, defined by increased exacerbations in respiratory pathology, reduced lung function, and increased mortality, particularly when infection becomes chronic [4]. Life-threatening infections with *P. aeruginosa* are also becoming increasingly frequent in patients with AIDS [5]. The coexistence of PJP and secondary bacterial infection complicates diagnosis and management due to overlapping clinical and radiological features [6].

Here, we present two illustrative cases of PJP in HIV-positive adults: one complicated by pseudomonal lung abscess in the setting of post-tubercular lung disease and advanced immunosuppression and another occurring with moderate immunosuppression and significant cardiopulmonary comorbidities.

## Case presentation

Case 1

A 38-year-old male laborer presented with a 15-day history of cough with expectoration, breathlessness on exertion, hemoptysis (3-4 episodes), on-and-off fever, and painful oral ulcers and loss of appetite. He had a significant past history of extrapulmonary tuberculosis treated 12 years earlier and was a known case of HIV infection diagnosed five years prior. He had been on antiretroviral therapy (abacavir/lamivudine/dolutegravir) but reported the discontinuation of treatment for 15 days before admission. He also gave a history of chronic alcohol consumption.

**Figure 1:**
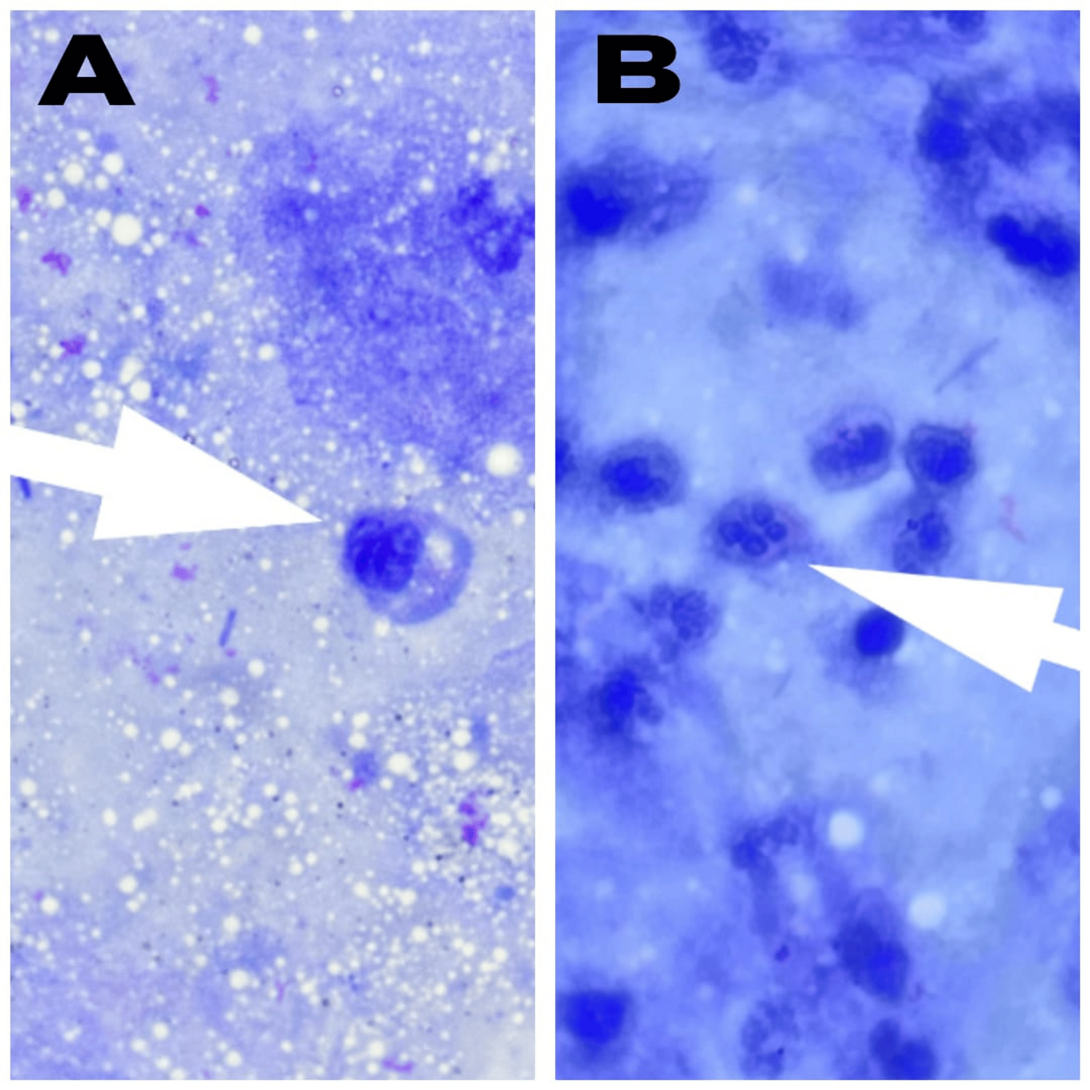
Case 1: Pneumocystis jirovecii cysts on Giemsa-stained sputum smear These cysts are round to oval bodies, approximately 5-8 µm in size, with a distinct foamy internal appearance typical of this organism (indicated by white arrows in both images A and B)

On examination, the patient appeared pale and dehydrated. His vital parameters were as follows: heart rate of 100/minute, blood pressure of 90/60 mmHg, respiratory rate of 28/minute, and oxygen saturation of 98% on room air. Oral cavity examination revealed whitish plaques consistent with oral candidiasis. Systemic examination demonstrated coarse crepitations in the right lower lung fields. Peripheral smear revealed microcytic hypochromic red cells with anisopoikilocytosis. Blood cultures were sterile. Microbiological evaluation demonstrated *Pneumocystis jirovecii *cysts on Giemsa-stained sputum smear (Figure 1).

The Gram stain showed a large number of polymorphs and slender Gram-negative bacilli. There was *Pseudomonas aeruginosa* growth on aerobic culture of sputum. On the antibiotic susceptibility report, it was sensitive to cefepime, ceftazidime, meropenem, aztreonam, ciprofloxacin, and piperacillin/tazobactam. Acid-fast bacilli smear and cartridge-based nucleic acid amplification test (CBNAAT) for *Mycobacterium tuberculosis* were negative. Other hematology and biochemical investigations are shown in Table 1.

**Table 1:** Laboratory investigations of Case 1 CD4: cluster of differentiation 4

Serial Number	Laboratory Parameters		Result	Reference Range	Interpretation
1	Hemoglobin	5.8 g/dL		13.5-17.5 g/dL (Male)	Severe Anemia
2	Total Leukocyte Count	18,000/mm³		4,000-11,000/mm³	Neutrophilic Leukocytosis
3	Platelet Count	3.27 × 10⁵/µL		1.5-4.5 × 10⁵/µL	Reactive Thrombocytosis
4	Sodium	125 mEq/L		135-145 mEq/L	Hyponatremia
5	Potassium	2.6 mEq/L		3.5-5.0 mEq/L	Hypokalemia
6	Albumin	1.9 g/dL		3.5-5.0 g/dL	Severe Hypoalbuminemia
7	Urea	66 mg/dL		15-40 mg/dL	Prerenal Azotemia
8	CD4 Count	80 Cells/mm³		500-1,500 Cells/mm³	Advanced Immunosuppression
9	C-reactive Protein	79 mg/L		<10 mg/L	Marked Inflammation

**Figure 2:**
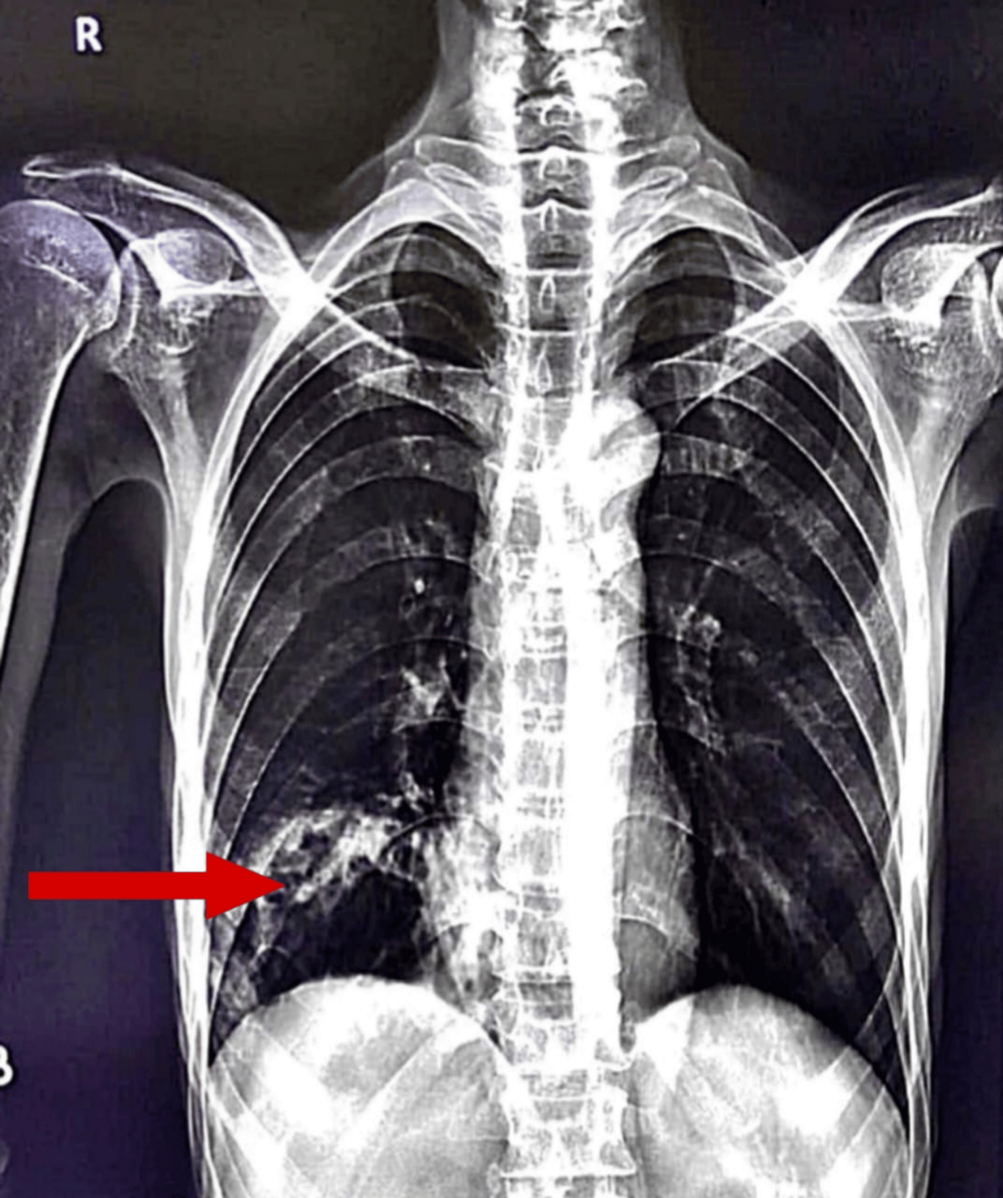
Case 1: posteroanterior view of chest X-ray showing right homogenous opacity in the right hemithorax involving mid and lower zones (red arrow)

Radiological investigations showed dense heterogeneous opacity in the right mid and lower lung zones with the obliteration of the right heart border and diaphragmatic outline on chest radiograph (Figure 2).

High-resolution computed tomography (HRCT) of the thorax revealed a large thick-walled fibrocavitary lesion involving the posterobasal segment of the right lower lobe with an air-fluid level within, with pulmonary changes of active infective etiology superimposed over sequelae of old infective etiology (Video 1).

**Video 1:** High-resolution CT of the thorax of Case 1 High-resolution CT of the thorax demonstrating multiple ground-glass and centrilobular nodular opacities with tree-in-bud appearance in the right middle and lower lobes, with associated cystic bronchiectasis. A large thick-walled fibrocavitary lesion with an air-fluid level is seen in the posterobasal segment of the right lower lobe. Multiple calcified mediastinal lymph nodes and splenic granulomas are also noted, consistent with sequelae of prior infection superimposed with active infective pathology CT: computed tomography

A diagnosis of advanced HIV infection with *Pneumocystis jirovecii* pneumonia, right lower lobe lung abscess, oral candidiasis, and sequelae of old extrapulmonary tuberculosis in a chronic alcohol user was established.

The patient was continued on antiretroviral therapy (tablet abacavir 600 mg + tablet lamivudine 300 mg + tablet dolutegravir 50 mg) and initiated on antimicrobial treatment comprising intravenous piperacillin/tazobactam (2.25 g three times daily), intravenous ciprofloxacin (200 mg twice daily), and oral trimethoprim-sulfamethoxazole (TMP-SMX) (Septran DS, once daily) during hospitalization. Tablet fluconazole 150 mg once daily was prescribed for oropharyngeal candidiasis. Supportive management included intravenous fluids, along with the continuation of the existing ART regimen.

After eight days of inpatient therapy, he was discharged on oral ciprofloxacin (500 mg twice daily for five days) and high-dose TMP-SMX (1.5 tablets thrice daily for 21 days) for the treatment of *Pneumocystis jirovecii* pneumonia. Adjunctive corticosteroid therapy with prednisolone was prescribed at 40 mg once daily, tapered to 20 mg once daily over 11 days. Hematinics were added in the form of folic acid (5 mg daily for 21 days) and pyridoxine (40 mg daily). The lifelong continuation of ART was reinforced.

Additional supportive measures included nutritional supplementation, fluid replacement, and the correction of electrolyte disturbances (hyponatremia and hypokalemia) and hypoalbuminemia. The patient demonstrated progressive clinical and radiological improvement during hospitalization and was discharged in stable condition on maintenance ART. He received intensive counselling regarding adherence to therapy and was advised on secondary PJP prophylaxis, along with regular follow-up in the HIV care clinic.

Case 2

A 55-year-old female homemaker, a known case of HIV infection on regular antiretroviral therapy for two years, presented with complaints of progressive breathlessness of one and a half months. The breathlessness was initially exertional but gradually progressed to occur even at rest and was associated with orthopnea. She also reported a productive cough during this period, along with retrosternal chest pain radiating to the left shoulder and associated tingling sensation. She further complained of loss of appetite for the past month. Her past medical history was significant for systemic hypertension and type 2 diabetes mellitus, for which she was on treatment. There was no history of recent tuberculosis treatment, occupational exposure, or travel.

On admission, the patient was dyspneic, tachypneic with a respiratory rate of 32/minute and blood pressure of 110/70 mmHg, and hypoxemic with oxygen saturation of 87% on room air. She showed the use of accessory muscles of respiration. Cardiovascular system examination suggested features of pulmonary arterial hypertension and ischemic heart disease. No hepatosplenomegaly or peripheral lymphadenopathy was noted.

**Figure 3:**
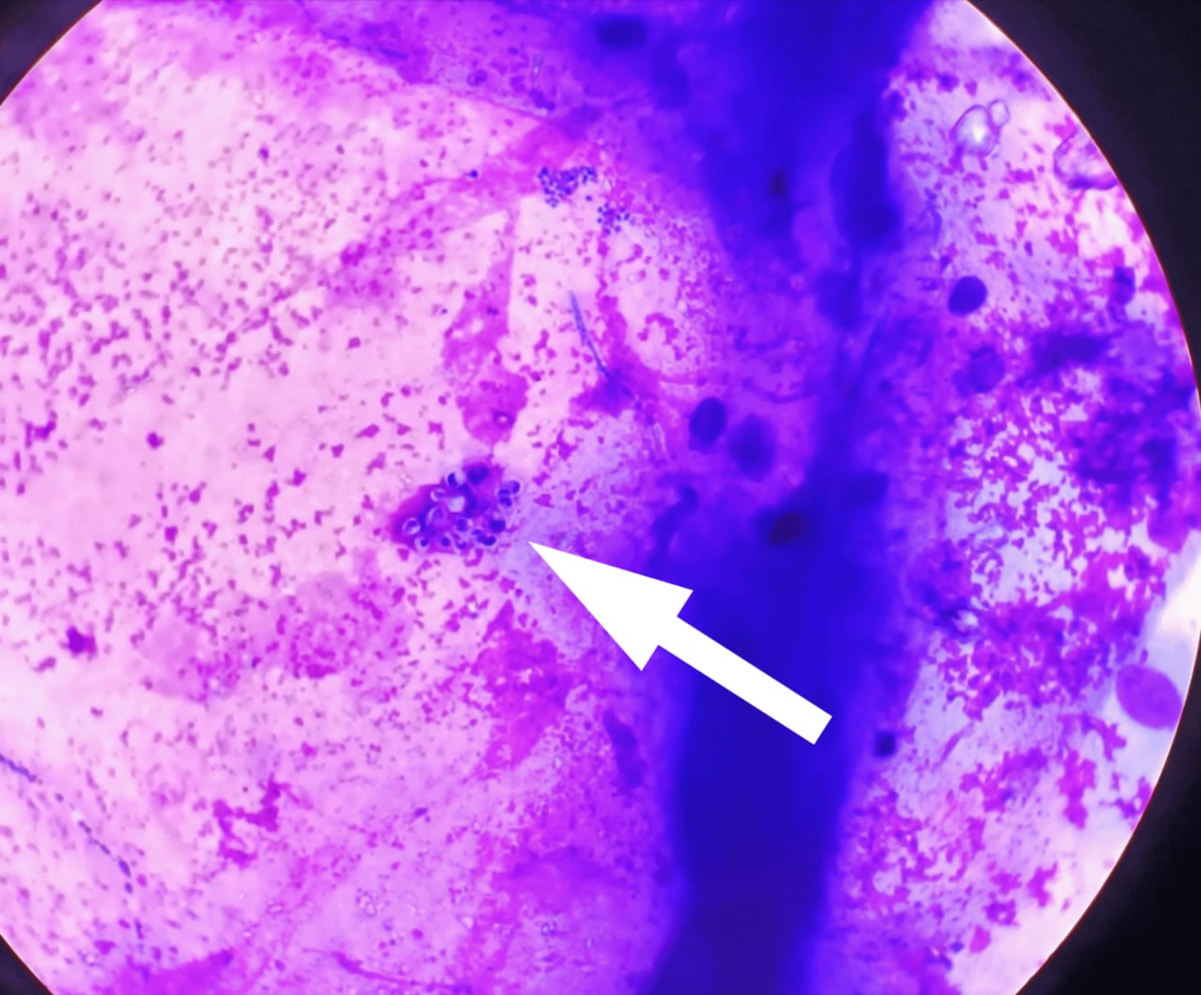
Case 2: Pneumocystis jirovecii trophic forms on Giemsa-stained sputum smear Wright-Giemsa-stained smear showing *Pneumocystis jirovecii* trophic forms (indicated by the white arrow). The trophozoites are small (1-4 µm), pleomorphic, and irregularly shaped with a prominent nucleus

Sputum examination with Giemsa stain demonstrated the presence of *Pneumocystis jirovecii* trophic forms (Figure 3), while bacterial cultures were negative.

Hematology and biochemical investigations are shown in Table 2.

**Table 2:** Laboratory investigation of Case 2 CD4: cluster of differentiation 4

Serial Number	Parameter	Result	Reference Range	Interpretation
1	Hemoglobin	13.8 g/dL	12-16 g/dL (Female)	Normal
2	Total Leukocyte Count	5.8 × 10³/µL	4.0-11.0 × 10³/µL	Normal
3	Platelet Count	1.43 × 10³/µL	1.5-4.5 × 10⁵/µL	Thrombocytopenia
4	CD4 Count	283 Cells/µL (15%)	500-1,500 Cells/µL	Moderate Immunosuppression
5	Urea	32 mg/dL	15-40 mg/dL	Normal
6	Creatinine	0.9 mg/dL	0.6-1.2 mg/dL	Normal
7	Sodium	149 mEq/L	135-145 mEq/L	Mild Hypernatremia
8	Potassium	3.35 mEq/L	3.5-5.0 mEq/L	Mild Hypokalemia
9	Total Protein	5 g/dL	6-8 g/dL	Hypoproteinemia
10	Albumin	2.9 g/dL	3.5-5.0 g/dL	Hypoalbuminemia

**Figure 4:**
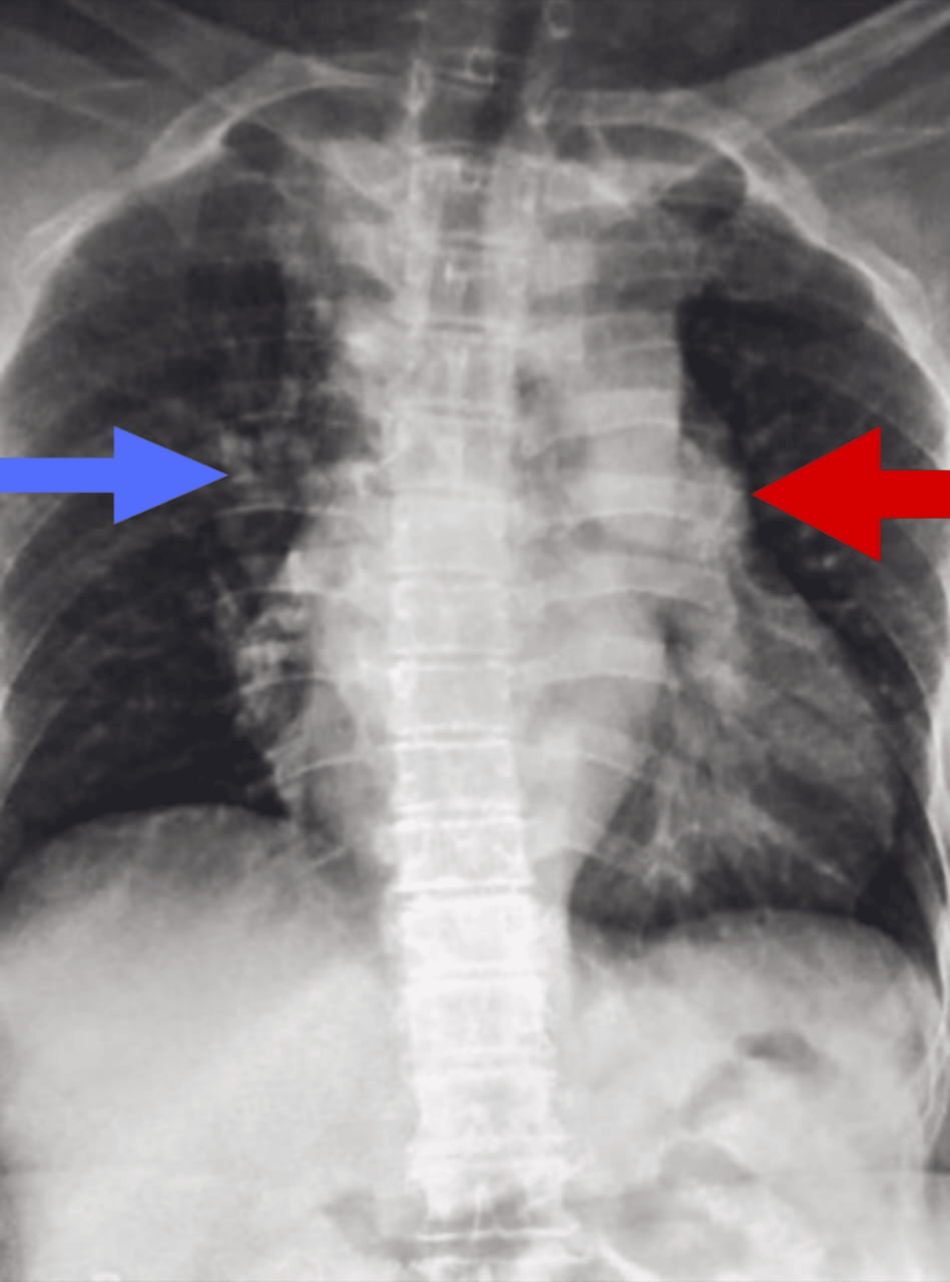
Case 2: posteroanterior view of chest X-ray showing right homogenous opacity in the right hemithorax involving mid and lower zones (blue arrow) The radiograph demonstrates a large, thick-walled cavitary lesion with an air-fluid level occupying the right lower lung zone, consistent with a fibrocavitary process. Surrounding parenchymal opacities and volume loss are noted in the adjacent lung fields with cardiomegaly (red arrow)

Chest radiography revealed bilateral patchy perihilar and peripheral opacities, along with cardiomegaly (Figure 4).

Arterial blood gas analysis was consistent with type 2 respiratory failure and metabolic acidosis.

Based on the above findings, a diagnosis of *Pneumocystis jirovecii* pneumonia in a patient living with HIV with type 2 respiratory failure, severe pulmonary arterial hypertension, systemic hypertension, type 2 diabetes mellitus, ischemic heart disease, and metabolic acidosis was made.

The patient was continued on her antiretroviral regimen consisting of abacavir (600 mg), lamivudine (300 mg), and dolutegravir (50 mg). During hospitalization, she received intravenous ceftriaxone (1 g), oral doxycycline (100 mg), TMP-SMX (Septran DS), telmisartan (2 mg), cilnidipine (1 mg), dapagliflozin (10 mg), and a fixed-dose combination of rosuvastatin (10 mg) with aspirin (75 mg) once daily. In addition, she was prescribed oral fluconazole (150 mg), prednisolone (40 mg), and folic acid (5 mg).

On discharge, she was initiated on high-dose trimethoprim (160 mg) with sulfamethoxazole (800 mg) for 21 days for the treatment of *Pneumocystis jirovecii* pneumonia. Adjunctive corticosteroid therapy was administered in the form of prednisolone 40 mg twice daily for five days, followed by 40 mg once daily for the next 10 days.

Supportive management included oxygen supplementation, the correction of electrolyte imbalances, nutritional support, and the optimization of comorbid conditions with appropriate antihypertensive, antidiabetic, and cardioprotective therapy.

The patient showed gradual clinical improvement with a reduction in dyspnea and hypoxemia. Follow-up chest radiography demonstrated partial resolution of pulmonary infiltrates. She was discharged on ART continuation with advice on strict adherence, regular follow-up, and monitoring of CD4 counts.

## Discussion

*Pneumocystis jirovecii* pneumonia remains one of the most common and life-threatening opportunistic infections in HIV, despite advances in antiretroviral therapy (ART) and prophylaxis. It is associated with significant morbidity and mortality in immunosuppressed individuals, particularly when diagnosis or treatment is delayed [7].

The initiation of ART in patients with PJP may precipitate immune reconstitution inflammatory syndrome (IRIS), a paradoxical worsening of symptoms and radiological findings due to an exaggerated host inflammatory response to residual *P. jirovecii *antigens [8]. This condition can mimic treatment failure or new infection, making recognition challenging. Clinicians must therefore carefully distinguish PJP-IRIS from antimicrobial resistance or co-infections to avoid unnecessary changes in therapy.

Unlike other fungi, *P. jirovecii* cannot be cultured in standard laboratory systems. Thus, the microscopic identification of organisms in respiratory specimens remains the diagnostic gold standard, utilizing stains such as Giemsa stain [9]. This limitation underscores the need for early specimen collection and the careful integration of microbiology with clinical and radiological data.

A CD4+ T-cell count of <200 cells/µL is the strongest predictor for PJP, and this threshold remains the basis for prophylaxis initiation [10]. However, PJP may also occur at higher CD4 counts. Reports document cases at counts of >400 cells/µL, often linked to high viral loads or additional comorbidities [11]. Indeed, about 5% of cases occur above 200 cells/µL [12]. Our patients illustrate this spectrum: one with advanced immunosuppression (CD4: 80 cells/µL), and the other with PJP despite a moderate CD4 count (284 cells/µL). These findings stress the importance of maintaining diagnostic vigilance even when CD4 counts exceed traditional cutoffs.

TMP-SMX remains the cornerstone of PJP treatment, with a recommended 21-day course for both HIV-infected and uninfected patients [9]. In our cases, both patients received this regimen, including the second patient whose CD4 count was above 200 cells/µL. Prophylaxis with TMP-SMX should be started when CD4 counts drop below 200 cells/µL in the setting of a detectable viral load [12]. Secondary prophylaxis is generally lifelong after PJP, though discontinuation may be considered in carefully selected patients with immune recovery and sustained virologic suppression [12].

Post-tubercular lung damage, including cavitation and bronchiectasis, predisposes to recurrent bacterial colonization. *Pseudomonas aeruginosa* is particularly problematic in this context, contributing to exacerbations and long-term decline in lung function [13,14]. In our first case, *Pseudomonas* infection in the setting of cavitary disease highlighted the impact of structural lung damage as a key driver of secondary bacterial infections and the need for tailored antipseudomonal therapy.

Beyond immunosuppression, factors such as malnutrition, anemia, electrolyte imbalances, and micronutrient deficiency further impair host defense [15]. Protein-energy malnutrition and hypoalbuminemia blunt T-cell function and delay recovery, while electrolyte disturbances increase physiologic stress. These findings emphasize that management must extend beyond antimicrobial therapy to include nutritional support, the correction of metabolic derangements, and adherence to ART. Tenofovir disoproxil fumarate (TDF), a nucleotide reverse transcriptase inhibitor (NRTI), is widely used as a first-line ART regimen in resource-limited settings such as India [16]. TDF can cause renal proximal tubular dysfunction and reduce the estimated glomerular filtration rate at a much higher level compared to other NRTIs such as abacavir (ABC), lamivudine (3TC), and zidovudine (AZT) [17]. In the present study, instead of the tenofovir + lamivudine + dolutegravir regimen, the patient was given the abacavir + lamivudine + dolutegravir regimen to prevent nephrotoxicity.

The two cases demonstrate distinct spectrums of PJP in PLHIV: one with severe immunosuppression complicated by post-TB lung disease and *Pseudomonas* infection and the other with PJP arising despite a moderate CD4 count. Together, they highlight key lessons: PJP can occur across a wide range of CD4 counts, IRIS should be considered after ART initiation, microbiological confirmation remains essential, and comprehensive management requires addressing co-pathogens, nutrition, and adherence, alongside standard antimicrobial therapy.

Limitations

This case series has a few limitations. Since it includes only two patients, the findings cannot be generalized to all people living with HIV. The diagnosis of *Pneumocystis jirovecii* pneumonia was based mainly on clinical symptoms, microscopy, and radiological findings, as confirmatory tests such as bronchoalveolar lavage with polymerase chain reaction (PCR) or β-D-glucan assays, as well as HIV viral load, were not available.

## Conclusions

In PLHIV with pulmonary symptoms, PJP should be considered even in moderate immunosuppression, particularly when complicated by structural lung disease or comorbidities. The coexistence of PJP and *Pseudomonas aeruginosa* infection poses diagnostic and therapeutic challenges. Early microbiological diagnosis, comprehensive imaging, and multidisciplinary management, including targeted antimicrobial therapy, supportive care, nutritional rehabilitation, and ART optimization, are crucial to improving survival.
